# A Modular Agentic Framework for Synthetically Constrained Multi-Objective Hit-to-Lead Optimization

**Published:** 2026-08-11

**Authors:** Kelvin P. Idanwekhai, Enes Kelestemur, Benjamin Strickland, Matthew Hart, Steini Davidsson, Angelos Angelopoulos, Ron Alterovitz, Marcello DeLuca, Alexander Tropsha

**Affiliations:** 1 Department of Chemistry, UNC Chapel Hill, Chapel Hill, NC, USA; 2 Division of Chemical Biology and Medicinal Chemistry, Eshelman School of Pharmacy at UNC, Chapel Hill, NC, USA; 3 Department of Applied Physical Sciences, UNC Chapel Hill, NC, USA; 4 Department of Computer Science, UNC Chapel Hill, Chapel Hill, NC, USA

## Abstract

Hit-to-lead optimization requires iterative design of hit analogs across competing potency, selectivity, physicochemical, pharmacokinetic, safety, and synthetic constraints. We present SABLE (Synthetically-accessible Agentic Bayesian Ligand Exploration), an open-source framework that employs natural-language orchestration to guide chemical structure optimization. SABLE uses an LLM to interpret user-defined goals and route tasks, while specialized tools perform reaction-templated analog enumeration, physicochemical and ADMET property prediction, structure-based affinity scoring, and Bayesian optimization. The resulting workflow is a computational twin of the analytical and prioritization stages of the design–make–test–analyze cycle, providing provenance of each numerical output. Across single, and multi-objective optimization studies, SABLE enriches candidate sets for user-defined computational objectives while evaluating only a subset of the enumerated search space. Its modular architecture allows tools and characterization backends to be replaced by editing a simple config file, without modifying operational logic. SABLE provides an extensible decision-support framework for prioritizing synthetically constrained analogs in early-stage drug discovery.

## 1 Introduction

Hit-to-lead optimization in medicinal chemistry proceeds through iterative design–make–test–analyze (DMTA) cycles in which medicinal chemists design, synthesize, and evaluate analogs of initial hits. The objective is not simply to maximize target potency, but to develop a chemical series that combines potency and selectivity with suitable physicochemical properties, pharmacokinetics, safety, and synthetic tractability. Each experimental optimization round expands the structure–property relationships that inform the next set of compounds[1]. However, finite synthetic and assay capacity limits evaluation to only a small fraction of the accessible chemical analog space, often requiring multiple chemical series to be advanced in parallel. Candidate selection must therefore account for both the immediate benefit of a structural modification and its compatibility with continued optimization. The latter challenge is further complicated by the need to optimize molecular structure not only for efficacy but also safety and drug-like properties that contribute substantially to clinical attrition[2].

This chemical structure optimization task is inherently multi-objective, and it is further complicated by strong interdependency between molecular properties. A structural modification intended to improve potency may also alter selectivity, lipophilicity, solubility, permeability, metabolic stability, or toxicity, and these effects cannot always be inferred independently. For example, matched molecular-pair analyses have shown that the same local transformation can produce different, and sometimes opposing, effects on hERG inhibition, solubility, and lipophilicity depending on its surrounding molecular context[3]. At the same time, potency, absorption, distribution, metabolism, excretion, and safety requirements frequently conflict at the campaign level[4]. Historical analyses have additionally cautioned that potency gains are often accompanied by increases in molecular size or lipophilicity, a tendency described as molecular inflation or molecular obesity[5]. Thus, as stated above, the central challenge is to develop multi-objective optimization strategies that can systematically balance competing requirements and guide compound selection toward an improved overall profile without introducing liabilities that must be corrected in subsequent rounds.

Machine learning methods are well suited to address this computational chemistry optimization challenge because they can evaluate large candidate sets consistently across multiple objectives. For instance, generative machine learning employing latent-space molecular generators, sequence-based SMILES generation, and LLM-driven molecular design[6–8] methods can propose large numbers of chemically valid structures with the desired properties[9]. Such methods can be devised not merely to generate plausible molecules, but to define an experimentally relevant search space and prioritize candidates within it according to explicit, competing objectives.

Many of the components required to construct such a workflow are readily available. Reaction-aware generation and enumeration methods can restrict molecular design to structures assembled from specified building blocks through encoded transformations[10, 11]. Property-prediction platforms and cheminformatics models provide calculated descriptors or model-based estimates for physicochemical, pharmacokinetic, and toxicological endpoints[12–14]. Structure-based models such as Boltz-2 jointly predict biomolecular complexes and affinity-related scores from protein-sequence and ligand inputs[15]. Bayesian optimization (BO), in turn, provides a principled framework for sequential decision-making when characterization is expensive and the relationship between structure and optimization objective is unknown[16]. By balancing exploration of uncertain regions with exploitation of candidates predicted to perform well, BO provides a natural mechanism for navigating a chemically constrained, multi-objective search space. The remaining difficulty is therefore less the absence of computational capabilities but the lack of a unified mechanism for coordinating them. At present, these capabilities remain distributed across standalone web services[12, 13], and locally installed cheminformatics toolkits[17–19]. Although individually capable, these resources demand nontrivial expertise to install individual modules, integrate them into a pipeline, and interpret the results of calculations, making such assembly, or a toolkit, difficult to use by the practicing medicinal chemist without dedicated informatics support. Any integrated system, therefore, must fulfill two distinct demands: it must be usable by the medicinal chemists whose design needs the tools are meant to support; and it must remain open to advances that will supersede today's components. AutoLead proposed a framework that combines LLMs and BO for lead optimization, but this workflow is limited by the modularity and type of oracle used[20]. The coverage of endpoints that can optimized by AutoLead, only include descriptors computed by RDKit[21], not accounting for other ADMET and potency properties. In addition, the framework lacks two important considerations: synthesizability-aware recommendation of molecules and modularity of workflow. The emergence of standardized model–tool interfaces, including the Model Context Protocol[22], provides a potential technical foundation for connecting modular software components and enabling the incorporation of new tools as they become available. Standardized connectivity alone, however, does not determine how chemical hypotheses should be evaluated, how conflicting objectives should be reconciled, or which candidate should be selected for the next iteration, reinforcing the need for systems that are both accessible to practitioners and adaptable to evolving computational capabilities.

Agentic methods offer one approach to this coordination problem by allowing a language model to interpret a task and invoke appropriate external tools. ChemCrow was, arguably, the first system that established the value of grounding LLM responses in expert-designed cheminformatics tools, with chemist evaluators preferring tool-augmented outputs on nontrivial tasks[23]. Coscientist extended this paradigm to robotic synthesis[24], while ChemReasoner combined Monte Carlo Tree Search (MCTS) with LLM-guided exploration for catalyst design[25]. These recent examples demonstrated that language models can provide accessible interfaces for coordinating specialized chemical tools. They also raise a separate question regarding the appropriate division of function between the language model and the quantitative models it invokes. In these architectures, the LLM remains central to scientific planning or hypothesis generation, while external tools supply information, calculations, or experimental execution. This arrangement can become problematic when the language model is also expected to judge the correctness of those outputs or choose among quantitatively competing candidates. ChemCrow's own evaluation illustrates the cost. According to the authors of ChemCrow, when their evaluator LLM, powered by GPT-4, was asked to assess completions, it could not reliably distinguish clearly incorrect LLM outputs from tool-grounded responses[23]. This finding illustrates the potential risk of using language-model judgment as the sole basis for quantitative assessment of chemical output, which has also been echoed by previous works that evaluate the effectiveness of LLMs as oracles in chemical optimization tasks[26, 27].

Herein, we introduce SABLE (Synthetically-accessible Agentic Bayesian Ligand Exploration), a modular platform for early-stage hit-to-lead optimization through language-model orchestration of specialized modules supporting chemical design and optimization tasks (Fig. 1). The LLM interprets user-defined goals, extracts the parameters required to configure the campaign, invokes the appropriate tools, and organizes their outputs. Molecular enumeration, descriptor calculation, ADMET property prediction, optimization, affinity scoring, and candidate ranking are delegated to explicitly defined cheminformatics components. Starting from a user-provided seed compound, SABLE uses Hit Expansion to Advanced Leads using Enumerated Reactions (HEALER) to construct a reaction-constrained analog space from available building blocks[11] (Fig. S1), evaluates selected candidates using physicochemical, ADMET, and structure-based characterization tools including Boltz-2[15], and applies BO to identify candidates that improve or balance the specified objectives. The resulting workflow implements a computational analogue of the analytical and prioritization stages of the DMTA cycle but is intended to support, rather than replace, medicinal-chemistry review and experimental validation.

By separating natural-language orchestration from candidate molecule generation, quantitative assessment, and selection, SABLE makes the optimization objectives, originating tools, and selection criteria explicit and traceable. Its modular architecture further allows enumeration, optimization, characterization, and experimental backends to be incorporated or replaced without altering the central orchestration and optimization logic. SABLE leans towards a bring your own tools (BYOT) approach, allowing users to define their own tools and predictors in a simple configuration file. More broadly, SABLE establishes a general design pattern for scientific agents in which language models provide an accessible and user-friendly interface between researchers and computational tools, while domain-specific models and explicit optimization algorithms retain responsibility for quantitative scientific decisions.

## 2 Results and Discussion

### 2.1 Workflow execution and state management

SABLE operationalizes the architecture shown in Fig. 1 as a stateful workflow implemented using LangGraph[28] (Fig. S2). Each campaign is initialized with the seed molecule, protein target, optimization objectives, iteration limit, and batch size extracted from the user request. A shared state records the enumerated candidate library, previously evaluated molecules, returned characterization values, and optimization history, allowing information generated at one stage to be accessed by subsequent workflow nodes.

During each iteration, the Bayesian optimization node recommends a batch of unevaluated candidates, which are routed to the characterization tools associated with the specified objectives. The returned values are appended to the campaign history and used to update the Gaussian-process surrogate before the next batch is selected. Conditional routing continues this cycle until the iteration limit is reached, the predefined convergence criterion is satisfied, or the enumerated search space is exhausted. LangGraph checkpointing preserves intermediate workflow states, enabling interrupted campaigns to resume without repeating completed characterization steps.

### 2.2 Single-Objective Optimization

We first evaluated whether SABLE could optimize a single computational objective using human calcium/calmodulin-dependent protein kinase kinase 2 (CAMKK2; UniProt Q96RR4) as the target[29, 30]. Starting from a seed compound, HEALER generated a reaction-constrained analog library, from which Bayesian optimization iteratively selected candidates for Boltz-2 evaluation[15] (Fig. 2A).

Across the campaign, the best-observed predicted logIC50 improved by 1.03 log units relative to the seed, while the cumulative distribution of the 20 best-observed candidates plateaued after approximately five iterations (Fig. 2A). Evaluated compounds were distributed across the projected analog space, although most of the enumerated library remained unevaluated at campaign termination (Fig. 2B). Thus, SABLE prioritized candidates with improved values of the assigned computational objective while characterizing only a subset of the available library; this behavior is characteristic of BO acquisition functions, which balance exploration of uncertain regions with exploitation of promising candidates.

### 2.3 Dual-Objective Optimization

We next evaluated whether SABLE could prioritize candidates across two objectives by jointly optimizing Boltz-2-predicted affinity and a quantitative estimate of drug-likeness (QED)[31]. Across successive iterations, the evaluated candidates produced a set of nondominated solutions spanning different compromises between lower predicted logIC50 and higher QED (Fig. S3). Some candidates favored one objective more strongly, whereas others achieved more balanced profiles (Fig. S3). The resulting Pareto frontier accordingly provides a set of alternative candidates rather than a single optimum, allowing subsequent selection to reflect project-specific priorities. These results show that SABLE can extend beyond single-objective optimization to prioritize candidate sets across an explicitly defined multi-objective landscape. All campaigns beyond one objective are guided with the qLogNEHVI acquisition function[32, 33], to maximize the expected hypervolume improvement. This approach evaluates candidate molecules based on how much they are expected to expand the Pareto front relative to previously evaluated compounds.

### 2.4 Multi-Objective Optimization

To further test the framework under more realistic drug discovery constraints, we performed optimization experiments that simultaneously considered multiple properties: Binding affinity, QED, and CNS Activity. SABLE can optimize additional ADMET and physicochemical properties (SI Note S5). Across multiple iterations, SABLE progressively sampled the candidate library to identify molecules that occupy favorable regions of the objective space (Fig 3A – C). Early iterations emphasized exploration to establish an initial Pareto frontier, while later iterations concentrated on regions predicted to yield balanced improvements across several properties (Fig. 3D). The resulting enriched libraries contained molecules that simultaneously satisfied multiple drug-like criteria, illustrating the ability of SABLE to perform automated multi-parameter optimization within a chemically constrained search space. From the resulting Pareto set, we chose a single compound that satisfies our tradeoff and present it as the ‘best’. In this work, we prioritize higher affinity scores over other objectives.

### 2.5 Efficiency of exploring the chemical space

One of the major challenges in ligand discovery is the enormous size of accessible chemical space. Even modest combinatorial modifications of the scaffold can yield millions of potential analogs. Exhaustive evaluation of these molecules is computationally infeasible, particularly when structure-based affinity predictions are required. To examine whether SABLE could effectively select optimal molecules from a large sample, we benchmarked the optimization routine on one-million-compound libraries using QED as the objective because it could be calculated for every molecule in this library. While there is criticism on using QED as a benchmark to compare across optimization methods[34], our analysis is appropriate to study how the optimization method affects the time-to-best-compound within a large library[35, 36].Separate campaigns were conducted using libraries generated by similarity- and diversity-based sampling (Fig. S4). We found that the optimizer recovered the global maximum after three iterations in the similarity-sampled library and seven iterations in the diversity-sampled library. The efficiency gain becomes even more pronounced as the number of objectives increases, since multi-objective optimization rapidly becomes intractable under exhaustive screening. Quantitatively, across the three campaign classes (sections 2.2 - 2.4), SABLE evaluated only a fraction of the enumerated library before convergence, translating to a reduction in Boltz-2 inference calls relative to exhaustive screening. The relative savings in oracle or experimentation calls, grow approximately linearly with library size in the regime of 〈10^3^ to 10^6^).

### 2.6 Case study 1: Retrospective METTL3 lead optimization

We next evaluated SABLE in a retrospective medicinal chemistry setting using the METTL3 inhibitor campaign reported by Dolbois et al. [37] This case study is useful since the published series contains a clearly defined experimental trajectory that optimizes an early METTL3 hit into the potent and selective inhibitor UZH2, with a reported single-digit nanomolar biochemical IC50 and favorable cellular target engagement. We supplied SABLE with the starting compound from the paper, specified METTL3 as the target using UniProt ID Q86U44, and asked the system to minimize predicted binding affinity using our agentic workflow.

The starting compound was predicted by Boltz-2 to have an IC50 of 9.77 *μ*M, which is consistent with the reported low-micromolar experimental activity of the starting compound (7 *μ*M)[37]. For the published optimized compound UZH2, Boltz-2 predicted an IC50 of 31.6 nM, whereas the experimental TR-FRET IC50 was 5nM. Thus, Boltz-2 moves in the direction of increased predicted affinity. The distinction is important since Boltz-2 in SABLE only serves as a computational oracle for enrichment and not a substitute for measured biochemical potency. However, the results of this exercise illustrate that SABLE will assure success in cases when computed ligand scores correlate with experimental measurements.

Despite this limitation, the retrospective run illustrates the practical value of the agent. Without using any experimental feedback from the METTL3 campaign, SABLE identified a top-ranked analog with a predicted concentration of 102.33 nM (Fig. 4). Relative to the starting compound, this represents approximately a 95-fold decrease in predicted concentration from a single optimization run. The candidate was not experimentally tested, so the result is interpreted as a prospective prioritization rather than experimental validation. Nevertheless, it demonstrates the ability of SABLE to move from a low-micromolar starting point to a predicted near-100 nM candidate for a challenging target such as METTL3.

The comparison also highlights a limitation of the current implementation. SABLE did not rediscover the exact published lead compound. The HEALER library used in this run did not include the reaction template required to construct that final triazaspiro[5.5]undecan-2-one series. The search space definition, therefore, constrained which structural modifications were available to the optimizer. The system can efficiently prioritize molecules within the accessible chemistry it is given, but its performance is limited by the coverage of the enumeration engine and the calibration of the characterization model. Expanding the reaction-template set and incorporating SABLE into experimental DMTA campaigns are therefore expected to improve both the chemical reach and the reliability of future campaigns.

### 2.7 Validation case study 2: Optimization on Boltz-2 tested targets

Because SABLE currently uses Boltz-2 as its structure-based affinity oracle, a natural test is to ask whether SABLE can improve ligands on targets for which Boltz-2 itself reports target-level affinity validation. Notably, the universal performance of Boltz-2 across all targets has been questioned [38, 39], which is why we sought targets where Boltz-2 performed well to examine the effectiveness of SABLE in the absence of potential failure attributable to the scoring function. In this case study, we select a few targets that Boltz-2 can rank with useful signals and use SABLE to turn a weak compound from an experimental series into analogs that have the same oracle scores as competitive with, or better than, the best in that series.

We selected four targets from the Boltz-2 affinity-validation set that span distinct target classes: beta-secretase 1 (P56817, protease), carbonic anhydrase XII (O43570, lyase), ABL1 (P00519, kinase), and sphingosine-1-phosphate receptor 1 (P21453, GPCR). These targets were useful for this case study since Boltz-2 reports per-assay validation performance for each of them in its hit-to-lead affinity benchmark, with positive rank and linear correlations across all four targets [15]. We then used the ChEMBL assay data for each target to define a deliberately difficult starting point. For each assay, the censored-low-activity compound in the experimental series was used as the seed molecule, while the best ChEMBL compound in the same assay provided a reference point for what a strong ligand in that series looked like. The ChEMBL data was used only for seed and reference selection. SABLE was not given the SAR trajectory or experimental activity labels during optimization; the only optimization signal was the Boltz-2 affinity score returned for candidates selected from the enumerated analog library.

We report this case study directly in the original concentration units (Table 1 and Fig. 5). The ChEMBL columns show experimental IC50 values from the assay series, while the Boltz columns show predicted concentrations from the SABLE runs. Lower concentrations indicate stronger activity or predicted affinity. For censored ChEMBL records, the original bound is retained. Across all four targets, SABLE identified analogs with improved Boltz-predicted affinity relative to the starting compound. The largest predicted shift was observed for P56817, where the seed was predicted at 58.88 *μ*M and the best SABLE analog at 370 nM. O43570 and P21453 also showed substantial predicted shifts, from 400 nM to 19.95 nM and from 1.38 *μ*M to 74.44 nM, respectively. P00519 began from a seed that Boltz already scored as considerably stronger than its ChEMBL assay value, but SABLE still improved the predicted value from 97.72 nM to 52.97 nM.

The case study shows that SABLE can use Boltz-2 as an optimization oracle in the setting for which Boltz-2 was designed, that is, in ranking compounds within hit-to-lead chemical series. While SABLE relies on Boltz-2, which does not provide excellent correlation between measured and predicted affinities of top binders, it still converges on molecules that score more than two orders of magnitude better than the starting points. Starting from the experimentally weakest compounds in the ChEMBL assay series, SABLE enriched the local analog space for the molecules with substantially lower predicted concentration values from their seed compounds. The final predicted values are also in the same broad range as the experimental activities of the best ChEMBL reference compounds for P56817, O43570, and P21453. For P00519, the seed prediction was predicted to be much more active, exposing a limitation in using Boltz-2 as an oracle.

SABLE enriches compounds against the Boltz-2 objective, and not against unmeasured biochemical truth. The result therefore supports the intended role of SABLE as a prioritization engine for proposing analogs that should be considered for synthesis or secondary computational analysis, rather than as a replacement for experimental potency measurements. In an experimental closed-loop deployment, the same workflow could replace or augment the Boltz-2 characterization node with real assay results as they become available, allowing the optimizer to use real-life experimental data while retaining the same agentic orchestration and selection framework.

## 3 Discussion

A key architectural design in SABLE is the distribution of the respective contributions between LLMs, QSAR, and optimization tools. The LLM does not optimize molecules but translates human intent into machine-readable optimization configurations; resolving molecule names to canonical SMILES, preparing node-routing configurations, and surfacing results back to the user. It is never used to propose analogs, score affinity, or reason about ADMET liabilities. Each of those decisions is delegated to a purpose-built component with validated statistical or physical grounding: HEALER for synthetically constrained enumeration, Bayesian Backend (BayBE) for BO, Boltz-2 for structure-based affinity, and dedicated QSAR predictors for ADMET endpoints. This responsibility split is what distinguishes SABLE from other systems like ChemCrow or Coscientist that place the LLM at the center of scientific reasoning. This design is informed by evidence that LLMs can hallucinate chemically invalid structures, misreport physicochemical properties, and are biased toward molecular patterns overrepresented in their training corpus. A frequent critique of tool-augmented agents like SABLE is that they trade fluency of an end-to-end LLM for engineering complexity [40]. Our results show that this tradeoff is justified in a technical domain such as drug discovery. Across single- and multi-objective campaigns (Figs. 2–3), SABLE produced improvements over the seed compound on every tracked objective, and the resulting enriched compounds consisted entirely of molecules constructible from commercially available building blocks under known reaction templates. This means that the resulting compounds are directly actionable by medicinal chemistry teams, in contrast to generative outputs that must first be triaged for feasibility. The BO layer further means that useful enrichment is obtained after evaluating only a small fraction of the enumerated space (Fig. 2), and efficiency gains compounds as the number of objectives grows and exhaustive evaluation becomes combinatorially infeasible.

The computational chemistry tool development landscape moves quickly, making it impractical to hardcode a particular affinity predictor or QSAR model should be replaced as soon as a better model or tool become available. SABLE is therefore designed around a standardized tool-integration interface that treats characterization and enumeration backends as both interchangeable and extendable. Swapping Boltz-2 for another affinity model or augmenting the ADMET stack with newly published toxicity predictors requires no change to the orchestration graphs or optimization logic, only a node-level adapter. The same principle extends to the enumeration layer, where HEALER could be replaced or complemented by other tools with similar functionality such as SynFormer or reaction-based generative models as they mature. This modularity positions SABLE as a non-static workflow, a substrate on which the community can build successively stronger optimization pipelines (Fig S2). In addition, SABLE’s architecture makes it possible to wrap the graph around laboratory equipment via REST APIs or Model Context Protocol (MCP) endpoints, allowing characterization nodes to dispatch real synthesis and assay requests rather than purely computational predictions. Because SABLE’s optimization loop is already framed around the DMTA paradigm and treats each characterization as a black-box oracle, the transition from simulated to experimental measurements is primarily an engineering integration rather than an algorithmic change. This design choice helps bridge the computational hit-to-lead optimization with closed-loop autonomous laboratories as the underlying robotic infrastructure matures.

Several caveats currently limit the agentic workflow. First, every claim SABLE makes about a candidate molecule is downstream of a predictive model, and predictive models for affinity, permeability, and toxicity remain imperfect; a library that is “enriched” in Boltz-2 logIC50 is enriched against its limitations, not against experimental ground truth. This motivates the eventual coupling to laboratory automation, where real measurements can close the loop. Second, HEALER’s synthetic accessibility is a necessary but insufficient condition for true experimental tractability. HEALER, by design, provides existing building blocks along with a reaction template, but this information alone does not constitute an experimental protocol. Furthermore, HEALER is designed to produce synthetically similar compounds; it does not provide the opportunity to assess structurally similar but synthetically dissimilar compounds; in the future, we will add the capability to explore this important category of chemical modifications that do not consider synthetic closeness.

Third, the Gaussian-process surrogate scales polynomially with each evaluated compound, and while this is tractable for conservative batch sizes, very large search spaces and long campaigns will eventually require space or deep-kernel approximations.

## 4 Conclusions

SABLE is an open-source agentic platform for ligand optimization. It can act in the lead-optimization part of the drug discovery pipeline, and it does this by executing a computational analog of the DMTA cycle that mirrors modern medicinal chemistry campaigns. Across single and multi-objective experiments, SABLE consistently enriched libraries for predicted property, drug likeness, and other relevant physicochemical properties, producing frontiers of synthesizable molecules that improved on a user-provided seed compound while evaluating only a small fraction of the enumerated search space.

Two properties distinguish this workflow. First, every molecule SABLE proposes is makeable from commercially available building blocks through retrosynthetic reaction templates, which eliminates failures that arise when generative models yield synthetically infeasible molecules. Second, the agent is designed for extension: its tool-integration interface allows any component like an affinity predictor, an ADMET stack, or an enumerator to be swapped for newer and better tools without touching the orchestration logic. Together, these properties position SABLE as a durable substrate for hit-to-lead optimization rather than a snapshot of today’s best tools.

## 5 Methods

### 5.1 Argument Extraction

Every task on SABLE begins with an argument extraction after a prompt is sent. The argument extraction node employs a hybrid extraction mechanism that combines rule-based regex parsing with LLM-assisted interpretation. The LLM component can handle ambiguous or complex prompts, resolving molecule names to structures and inferring implicit optimization objectives. From a prompt, SABLE can extract the starting molecule (SMILES), protein target (UniProt ID), optimization target (binding affinity, minimize), enumeration size, iteration count, and batch size. Importantly, SABLE automatically detects the number and types of objectives present in the user prompt during the argument extraction state (Table S2). These objectives are passed to the Bayesian optimization node, which dynamically configures the optimization strategy to handle either single-objective or multi-objective problems without requiring user intervention. All prompt extractions were done with GPT-5-chat preview (version 2025–10-03), provided by Azure OpenAI.

### 5.2 Name to Entity

After argument extraction, SABLE runs a “name-to-entity” step to normalize user-specified molecular and protein identifiers into machine-readable structures. This module maps common compound/drug names (e.g., aspirin, ibuprofen) to SMILES strings, enabling downstream generation and scoring without requiring users to provide structures explicitly. For target proteins, it accepts UniProt IDs and retrieves the corresponding structural entities used by the optimization workflow.

To improve robustness, the node uses a hybrid resolver that combines deterministic identifier matching with LLM-assisted disambiguation for incomplete or ambiguous names. Retrieved molecules are standardized (e.g., canonicalized SMILES) before being passed to later stages, while protein entities are validated against available structure metadata. This ensures that both ligand and target inputs are consistently represented before Bayesian optimization begins.

### 5.3 Molecular search space enumeration

Ligand generation carries the requirements that the ligands are both synthesizable and rationally permutable from a starting compound – without these two features, evolution of ligands toward more optimal candidates cannot proceed. We incorporated HEALER as a molecular enumeration tool[11]. HEALER workflow contains three modes of molecule enumeration that serve slightly different purposes. Molecule-mode is the default way of generating synthesizable analogs of a given seed molecule. HEALER initially performs a recursive retrosynthetic fragmentation of the query molecule using predefined reaction templates to create a retrosynthesis tree with fragment and reaction annotations. In the next step, HEALER matches building blocks to fragments based on chemical similarity and recombines them via predefined reactions to produce analogs of the seed molecule. Similarly, Fragment-mode allows the enumeration to directly start from fragments and generate molecules that combine the given fragments in a synthetically feasible way. Lastly, site-mode allows the reaction enumerations to take place at specified reactive sites of the seed molecule while preserving the rest. In this study, Enamine US building block stock was used by HEALER, and Molecule-mode was allowed to have at most two retrosynthesis reaction steps to prevent long enumeration runtimes.

This approach addresses the molecular hallucination problem. Unlike generative models that propose arbitrary SMILES strings, every molecule in SABLE’s search space is constructed from these purchasable fragments. HEALER also accepts limits on the chemical similarity of evolved compounds, providing a tunable parameter to balance between fine-grained and coarse-grained exploration of the chemical space. Given the modularity of SABLE, the enumeration node is tool-agnostic; that is, any other molecular enumeration tool can be used in place of HEALER, such as SynFormer[10].

### 5.4 Optimization Node

Most agentic platforms for chemical discovery use LLMs to iteratively suggest chemical perturbations to improve the desired properties of a discovery campaign [23, 41, 42]. This is done under the assumption that LLMs can apply chemical rationale. While this can work for short-horizon and single-objective optimization, it is subject to significant bias arising from LLM training and may not adequately explore the chemical space when the task allows for broad exploration and sensible chemical modifications. Furthermore, multi-objective optimization is not tractable with this approach. We integrated an optimization node that allows SABLE to perform both single- and multiple-objective optimization of molecular properties. Bayesian optimization is used in this case; it is a sequential design method for globally optimizing black-box functions. In the case of lead optimization, a black-box function has no interpretable functional form and is hard to measure. Examples of these are binding affinity, toxicity, etc. Our Bayesian optimization module consists of a Gaussian process surrogate model and a *qLogExpectedImprovement(qLogEI)* acquisition function. In the case of multi-objective optimization, we utilize the *qLogNoisyExpectedHypervolumeImprovement (qLogNEHVI)* acquisition function.

The optimization node uses BayBE’s Campaign API to recommend the next batch of molecules for evaluation. Molecules are encoded as *SubstanceParamater* objects using one of several fingerprint schemes: Mordred, Extended Connectivity Fingerprints (ECFP), or RDKit descriptors. The surrogate model is updated with all prior experimental results at each iteration, and the acquisition function selects the most promising unevaluated molecules. The system validates recommendations against the search space and filters out previously tested molecules before forwarding them for characterization. Extended information about this node is provided in (SI Notes S3 – S5).

### 5.5 Characterization Nodes

SABLE integrates characterization backends that are selected automatically based on the optimization target of interest. **RDKit** computes molecular descriptors such as QED, LogP, TPSA, molecular weight, H-bond donors/acceptors, rotatable bonds, ring count, etc [21]. **STOPLIGHT** predicts physicochemical and structural properties, assay liabilities, and pharmacokinetic properties for small molecules [14]. **Boltz-2** accepts ligand SMILES and protein sequences (or UniProt IDs, resolved automatically) and returns affinity estimates. A *‘decide_characterization’* node in the agent’s graph inspects the target properties and selects the minimal set of tools required. If all targets are RDKit-computable, only RDKit is invoked. Binding affinity targets trigger Boltz-2. Properties spanning both toolsets invoke a combined pipeline.

### 5.6 Binding Affinity Scoring

Binding affinity prediction was performed using Boltz-2[15], a deep learning model for biomolecular structure and property prediction based on a diffusion-transformer architecture. Candidate molecules selected by the optimization module are provided to the model as SMILES strings, while the amino acid of the target protein is retrieved by the argument extraction tool. The Boltz-2 YAML input is prepared from the provided SMILES and target sequence information. Boltz-2 jobs are dispatched asynchronously to a dedicated inference backend. In API mode, the YAML payload is submitted via an authenticated HTTP request to a REST endpoint, and the client polls for jobs at intervals; all ligands within a given optimization batch are processed concurrently using asynchronous I/O. Alternatively, in Celery mode, jobs are submitted to a SLURM-managed high-performance computing cluster via a task queue, with execution performed inside an Apptainer container, enabling GPU-accelerated inference. Upon completion, the model returns an affinity prediction value extracted from the per-job JSON output. This value is recorded and passed back to the optimization module as the objective signal for subsequent molecule selection. The predicted 3D structure of the protein-ligand complex is additionally retrieved and stored as a CIF file, providing structural interpretability alongside the scalar affinity score.

### 5.7 Stopping Criteria

Optimization loops within SABLE terminate when one of several predefined conditions is met. These stopping criteria prevent unnecessary computation once the search process has converged or the search space has been exhausted. (i) Maximum number of iterations specified by the user has been reached. This parameter provides an upper bound on runtime and computational cost. (ii) The convergence node monitors improvement in the objective function across iterations. If no significant improvement is observed over several consecutive iterations, the system interprets this as convergence of the optimization process. (iii) The workflow terminates if all molecules within the enumerated search space have been evaluated. This condition typically occurs only when the search space is relatively small. (iv) Optimization stops if the remaining unevaluated molecules are fewer than the specified batch size. In this case, the system evaluates the remaining candidates and completes the campaign.

### 5.8 Webapp and user interface

SABLE is distributed with a CLI, but it also includes a web-based interface designed to simplify interaction with the optimization workflow. The interface allows users to submit prompts describing optimization objectives, seed molecules, and monitor the progress of active optimization campaigns. The backend server manages agent execution and dispatches tasks to the appropriate computational modules. Results from each iteration are stored in a database and streamed to the interface, allowing users to inspect intermediate results such as predicted affinities, molecular properties, and candidate structures. The visualization components allow the results to be inspected and downloaded. The full architecture is shown in Fig. S2.

### 5.9 Handling ambiguous questions

User prompts may contain ambiguous or incomplete information regarding optimization targets, molecule identity, or experimental constraints. If the prompt lacks certain parameters, the system uses default configurations. This process ensures that optimization campaigns begin with well-defined parameters while still allowing for cases where the user might not be familiar with optimization jargon. When critical information is missing, such as the starting molecule name or a protein target required for affinity prediction, the system fails early and informs the user of what they might need to provide.

### 5.10 Computational environment and configurations

All SABLE experiments reported in this text were performed in a Dockerized environment on a single laptop. The Docker containers ran on Ubuntu 22.04.5 LTS via the Windows Subsystem for Linux (WSL), restricted to 10 CPU cores and 32 GB of RAM. Binding affinity estimations with Boltz-2 were carried out on a compute node equipped with four NVIDIA L40 GPUs (46 GB each). Each protein–ligand affinity inference was carried out in an Apptainer (Singularity) environment with a single GPU.

## Supplementary Material

Supplement 1

## Figures and Tables

**Figure 1:**
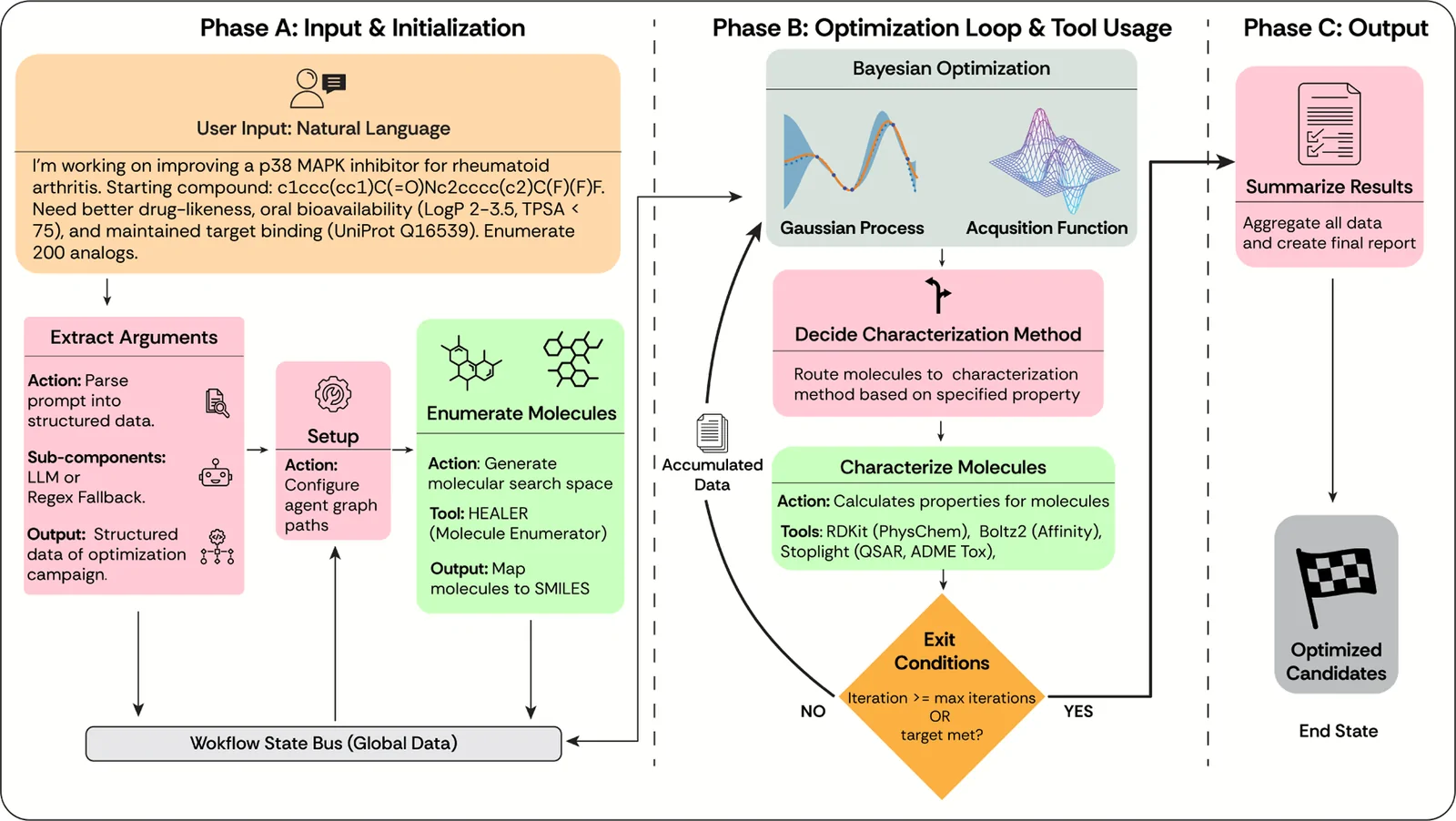
Schematic representation of the ligand optimization workflow. (A) A user prompts the system in natural language; then, key arguments for the optimization run, like the starting molecule, protein target, optimization objectives, and number of iterations, are extracted with LLMs. Analogs of the starting compound are also enumerated according to the user specification or default configuration. (B) An optimization campaign is initiated on enumerated compounds. Properties predicted with tools such as Boltz, RDKit, or STOPLIGHT are used in place of experimental measurements. This iteration is performed until predefined process termination criteria are met (see Section 4.7). (C) Optimized candidates are selected and reported to the user.

**Figure 2:**
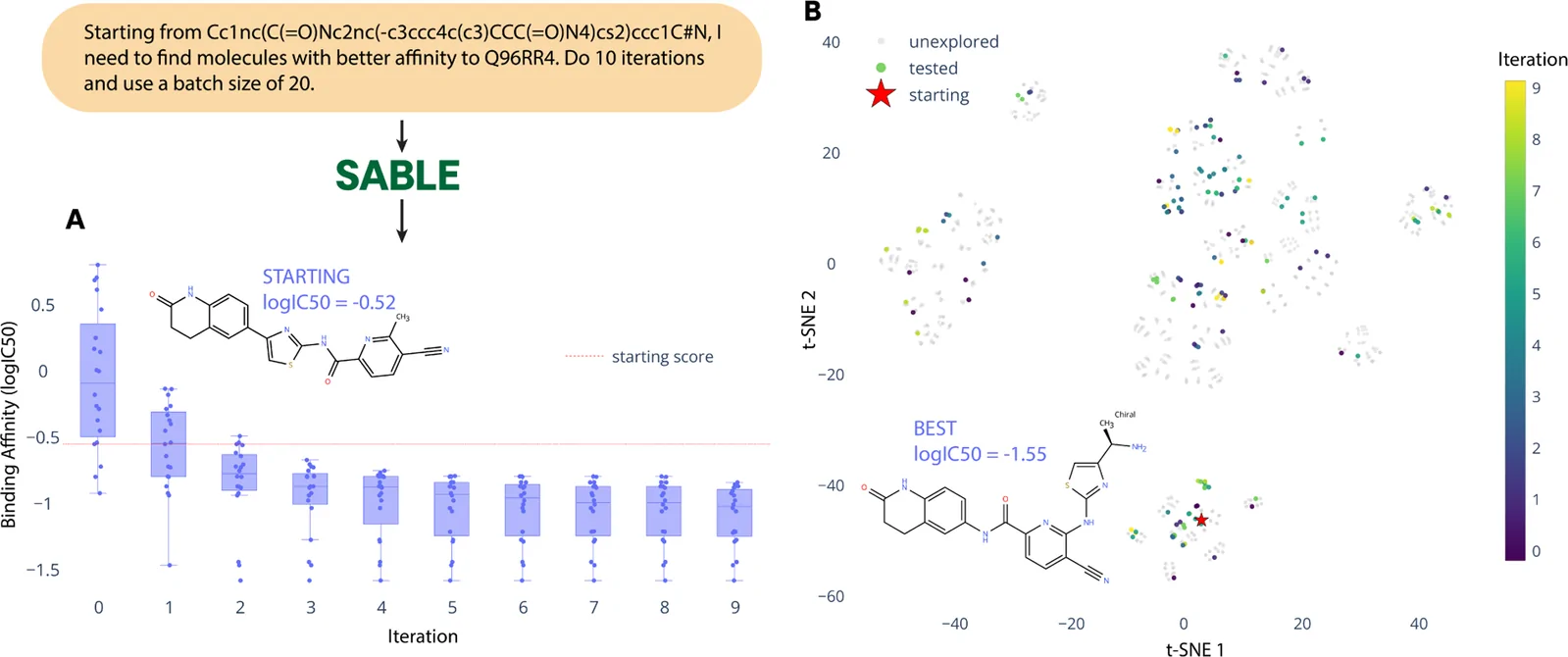
Single-objective optimization of binding affinity. Starting from a ligand specified by its SMILES string, SABLE enumerates analogs and iteratively finds molecules with higher affinity for the target with UniProt ID Q96RR4. (A) Box plots show the cumulative distribution of the 20 lowest predicted Boltz-2 scores (in logIC50). Lower values correspond to stronger predicted affinity. The red dotted line indicates the seed compound's logIC50 of –0.52; the best-observed candidate had a predicted value of –1.55. (B) t-SNE visualization of the enumerated analog space. Colored points indicate evaluated candidates, with color denoting the iteration in which each candidate was evaluated; grey points indicate candidates that remained unevaluated at campaign termination. The structure and predicted logIC50 of the best-observed candidate are shown.

**Figure 3:**
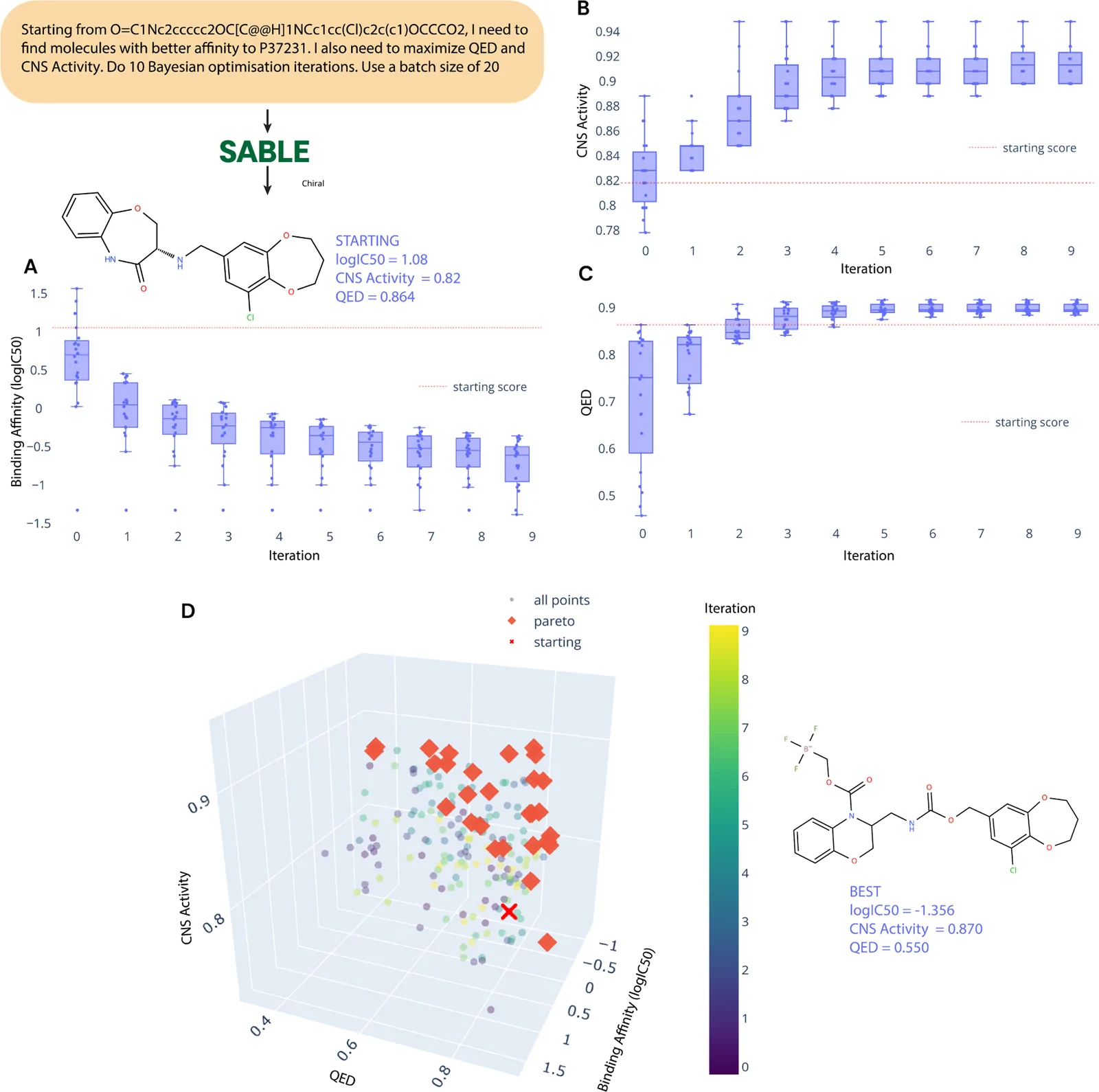
The multi-objective optimization of affinity, QED, and CNS activity. (A) Boxplots showing the affinity distribution in logIC50 of the best 20 ligands evaluated so far in the optimization campaign. The structure of the starting compound is shown with logIC50, CNS Activity, and QED scores of 1.08, 0.82, and 0.86, respectively. The red dotted line indicates the score of the starting compound. (B) Boxplots showing the distribution of the best 20 ligands that optimize the CNS activity objective. (C) Boxplots showing the distribution of the best 20 ligands that optimize the QED objective. (D) 3D plot showing the Pareto frontier of all three optimization objectives. The best compound resulting from the optimization campaign is also pictured. We observe improvements in binding and CNS activities, but a lower QED based on the tradeoff.

**Figure 4:**
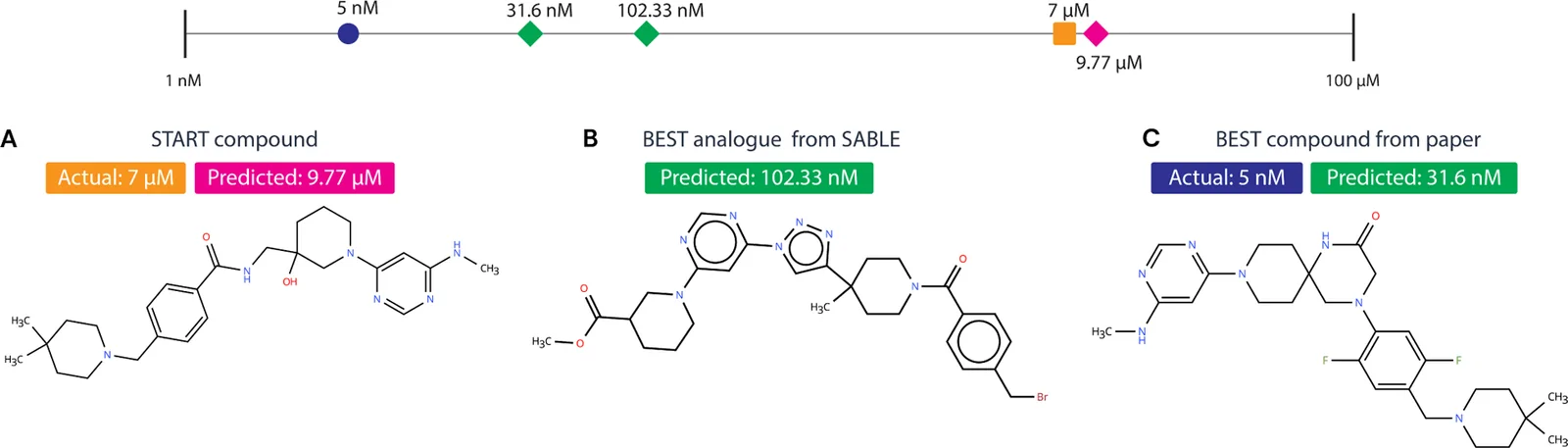
Retrospective METTL3 lead-optimization case study. (A) The starting compound is shown with both the reported experimental IC50 and the Boltz-2-predicted concentration. (B) The best SABLE analog is shown as a Boltz-2-predicted value since it has not been experimentally tested. (C) The published best compound is shown with both its experimental IC50 and Boltz-2-predicted concentration.

**Figure 5:**
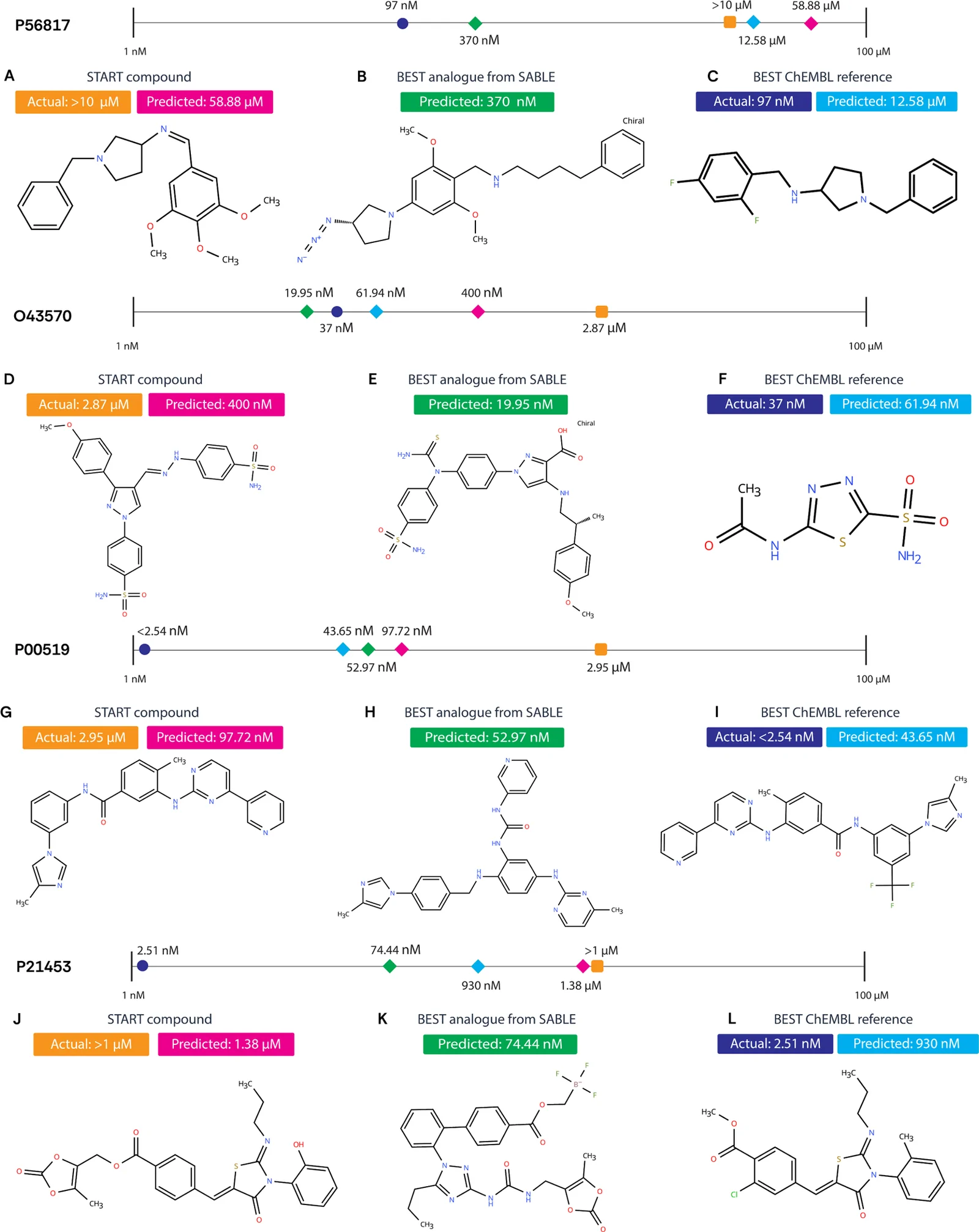
Ligand and affinity comparison for the retrospective Boltz-2 target validation. Each panel shows the ChEMBL seed compound selected from the weak end of the assay series, the best ChEMBL reference compound from the same series, and the best analog identified by SABLE. Lower concentration values indicate stronger activity or predicted affinity.

**Table 1:** SABLE optimization on Boltz-2 affinity-validation targets, reported in experimental and predicted concentration units

Target ChEMBL assay reference	ChEMBL value for seed molecule	Boltz-2 prediction for seed	Best ChEMBL reference	Boltz-2 prediction for ChEMBL best	Best from SABLE

P56817 Beta-secretase 1 / BACE-1 FRET assay	> 10 *μ*M	58.88 *μ*M	97 nM	12.58 *μ*M	370 nM
O43570 Carbonic anhydrase XII / spectrophotometric	2.87 *μ*M	400 nM	37 nM	61.94 nM	19.95 nM
P00519 ABL1 / peptide-substrate inhibition assay	2.95 *μ*M	97.72 nM	< 2.54 nM	43.65 nM	52.97 nM
P21453 S1PR1 / beta-arrestin recruitment assay	> 1 *μ*M	1.38 *μ*M	2.52 nM	930 nM	74.44 nM

## Data Availability

SABLE is released under an Open Source Apache license and can be found at https://github.com/molecularmodelinglab/SABLE. The Dockerfiles and Apptainer SIF reproducing the exact software stack are released alongside the code. The prompts and JSON state-dumps required to reproduce every figure are also deposited in the repository.
