# Supplementary material for: A Modular Agentic Framework for Synthetically Constrained Multi-Objective Hit-to-Lead Optimization
Source: ArXiv. 2026 Aug 11:arXiv:2608.11483v1. Preprint. [Version 1] (PMC13640889)
Supplement: Supplementary file 1 [file NIHPP2608.11483v1-supplement-1.pdf]

Supplemental Information

S.1 Molecule Encoding

Molecules entering the optimization campaign are wrapped as *SubstanceParameter* objects, a discrete label-like parameter type that maps human-readable molecule labels to canonicalized SMILES strings. Encoding from chemical structure to a numerical feature vector consumable by the Gaussian process surrogate proceeds in four stages.

Stage 1: SMILES validation and canonicalization. Each SMILES string supplied is canonicalized via RDKit, and duplicates are rejected. This guarantees a one-to-one correspondence between labels in the search space and points in the descriptor space, preventing degenerate rows in the kernel Gram matrix that would otherwise destabilize the posterior.

Stage 2: Fingerprint/descriptor computation. SABLE can use three encoding schemes through the BayBE *SubstanceEncoding* enum, selectable per campaign.

- MORDRED (default): a >1,600-dimensional vector of 2D molecular descriptors covering topological, constitutional, geometric, and electronic properties.
- ECFP (Extended-Connectivity Fingerprints)**:** radius-3 circular fingerprints folded into a 2048 length bit vector, capturing local atomic environments.
- RDKIT**:** the native RDKit descriptor set (~200 descriptors), spanning physicochemical properties such as logP, TPSA, and ring counts.

Stage 3: Cleaning. Once the raw descriptor matrix is computed, columns containing any *NaN* values and columns with zero variance are removed. These columns carry no information and would either crash the kernel computation or unnecessarily inflate the dimensionality.

Stage 4: Decorrelation. High-dimensional cheminformatics descriptors are heavily redundant. We therefore apply a correlation threshold filter, i.e., pairs of descriptors with correlation exceeding 0.7 are reduced to a single representative. As noted in the Bayesian Backend (BayBE) documentation, for libraries on the order of tens of thousands of molecules, this typically reduces descriptors to only informative features. This step is necessary for the Gaussian process since redundant features inflate the effective length-scale prior, slow the marginal-likelihood optimization, and degrade out-of-sample predictive variance. Finally, a small Gaussian perturbation is applied to any rows that remain numerically identical after decorrelation, ensuring the kernel matrix is strictly positive-definite.

The cleaned, decorrelated dataframe is cached and passed downstream as the feature representation $X \in \mathbb{R}^d$ for each candidate molecule.

S.2 Gaussian Process Surrogate

The surrogate is a SingleTask gaussian process wrapped by BayBE's *GaussianProcessSurrogate* class. Given training data $\mathcal{D} = \{(x_i, y_i)\}_{i=1}^{n}$, the model defines a prior

$$f(x) \sim \mathcal{GP}(m(x), \kappa(x, x')),$$

with constant function $m(x) = c$ and covariance kernel $\kappa(x,\ x')$ produced by the *DefaultKernelFactory.* The default factory adapts the kernel to the dimensionality and structure of the search space, defaulting to a Matérn 5/2 kernel with automatic relevance determination (ARD), one length-scale per input dimension:

$$K_\nu(x, x') = \frac{1}{\Gamma(\nu)2^{\nu-1}} \left(\frac{\sqrt{2\nu}}{\ell} d(x, x')\right)^\nu K_\nu \left(\frac{\sqrt{2\nu}}{\ell} d(x, x')\right)$$

where $d(x, x') = \|x - x'\|$ is the Euclidean distance, K_ν is the modified Bessel function of the second kind, and $\ell \in \mathbb{R}^d$ is the per-dimension length-scale vector inferred by maximum marginal likelihood. The Matérn 5/2 kernel produces sample paths that are twice mean-square differentiable, a smoothness assumption well matched to molecular property surfaces, which are continuous functions of descriptor space but typically not infinitely differentiable.

Likelihood. Observations are modeled as

$$y_i = f(x_i) + \varepsilon_i, \qquad \varepsilon_i \sim \mathcal{N}(0, \sigma_n^2),$$

with a *GaussianLikelihood.* The noise variance σ_n^2 is initialized and constrained by a data-driven prior produced, which sets a weakly informed LogNormal prior over σ_n^2 scaled to the spread of the target variables. This prior regularizes the noise estimate when n is small and prevents the marginal-likelihood optimizer from collapsing $\sigma_n^2 \ \rightarrow \ 0$

S.3 Acquisition Functions

SABLE uses logarithmic, batch (q-) Monte Carlo variants of expected improvement throughout. These are provided through the BayBE library. The log transformation reformulates expected-improvement acquisition functions to avoid numerical underflow in regions where the analytics improvement is small, which frequently occurs in late-stage lead optimization once the surrogate is well-trained[S1]. This improves the optimization of the acquisition surface relative to the standard qEI

S.3.1 Single-objective: qLogExpectedImprovement (qLogEI)

For a batch $X = \{x_1, \ ... x_q\}$ and observed best value $f\left(X^+\right)$, the standard batch expected improvement is

$$qEI(X) = \ E_{Y\sim p(Y|X,\ D)} \left[\max\left(0,\ \max_i y_i - \ f\left(X^+\right)\right)\right],$$

estimated by Monte Carlo with N samples drawn from the joint Gaussian process posterior:

$$qEI(X) \ \approx \ \frac{1}{N}\sum_{j=1}^{N}\max\left(0,\ \max_i y_i^{(j)} \ - \ f\left(X^+\right)\right).$$

qLogEI replaces the non-smooth $max(0, \ \ .)$ and the final $\max_i$ operators with their LogSumExp-based smooth analogs, computing log-EI directly in a numerically stable way:

$$qLogEI(X) \approx logsumexp_j \left[softplus_\tau\left(\max_i^\tau \ y_i^{(j)} - f\left(X^+\right)\right)\right] - logN,$$

where $softplus_\tau(z) \ = \ \tau^{-1}log(1 + exp(\tau z)$ and $\max^\tau$ is the temperature-τ smooth maximum. Critically, the gradient $\nabla_X \ qLogEI(X)$ remains well-conditioned even when EI itself has saturated to machine epsilon, allowing the multi-start L-BFGS-B optimizer over the acquisition to reliably locate batch optima that would be missed by qEI.

S.3.2 Multi-objective: qLogNoisyExpectedHypervolumeImprovement (qLogNEHVI)

For multi-objective optimization, SABLE uses qLogNEHVI. Given M objectives and a Pareto front $\mathcal{P}_\mathcal{D}$ inferred from the noisy training data, define the dominated hypervolume relative to a reference point r as

$$HV\left(\mathcal{P};r\right) = \ \lambda_M\left(\bigcup_{p\ \epsilon\ \mathcal{P}}[r,p]\right),$$

whereλ_M is the M-dimensional Lebesgue measure. The noisy expected hypervolume improvement (qNEHVI) integrates the hypervolume gain over both the posterior at the candidate batch *and* the posterior at the existing observations, marginalizing over noise on the inferred front:

$$qNEHVI(X) = \ \mathbb{E}_{\widetilde{\mathcal{P}}\sim p(.|\mathcal{D})}\mathbb{E}_{Y\sim p(Y|X,\mathcal{D})}\left[HV(\widetilde{\mathcal{P}}\cup Y;r) \ - \ HV(\widetilde{\mathcal{P}};r)\right].$$

qLogNEHVI applies the same LogSumExp smoothing as qLogEI to the hypervolume difference, again yielding smooth, non-vanishing gradients across the objective space and across the Pareto front. The reference point r is computed automatically from the observed objective values with a configurable safety margin, ensuring r is dominated by all observed points along every objective.

Table S1: Summary of different agentic systems chemistry and their design approach

System	Agent role	Candidate selection	Synthesizability constraints	Closed-loop with experiments
ChemCrow[S2]	LLM as scientist	LLM	Implicit	In theory
Coscientist[S3]	LLM as scientist	LLM + heuristics	Implicit	Robotic synthesis
ChemReasoner[S4]	LLM + MCTS	MCTS-LLM	None	No
**SABLE (this work)**	LLM as orchestrator	Bayesian optimization	Explicit (HEALER reaction templates + Enamine BBs)	Architected

S.4 Recommendation Loop

At every iteration k, the Bayesian optimization node executes the following procedure inside BayBe's *Campaign* API:

1. The surrogate model is updated by refitting the Gaussian process from scratch on the full set of measurements $\mathcal{D}_k$ accumulated so far (Section S.2).
2. The acquisition surface is constructed by instantiating qLogEI (single objective) or qLogNEHVI (multi-objective) with the updated posterior (Section S.3).
3. Optimization is done by maximizing the acquisition value over the unevaluated portion of the search space using a batch acquisition optimizer to obtain a candidate batch X_k of size q.
4. Each recommendation is cross-checked against the original search space (to enforce constraints and the substance label set) and against the historical ledger (to filter molecules already characterized in any prior iteration).
5. The validated X_k is forwarded to the appropriate characterization node, and the returned measurements are appended to $\mathcal{D}_{k+1}$, while the loop repeats.

The de-duplication step in (4) is essential because the Gaussian process posterior near previously observed points has very low variance, an unconstrained acquisition optimizer would otherwise be free to recommend arbitrarily small perturbations of already-tested compounds. Filtering against the experiment ledger forces the recommended batch to occupy genuinely new regions of chemical space, preserving the explore-exploit trade-off that motivates the use of Bayesian optimization in the first place.

S.5 List of Properties Currently Supported by SABLE

RDKIT_PROPERTIES = {

"molecular_formula", "exact_mass", "molecular_weight", "heavy_atom_count",

"ring_count", "aromatic_ring_count", "h_bond_donors", "h_bond_acceptors",

"rotatable_bonds", "logp", "tpsa", "qed"

}

STOPLIGHT_PROPERTIES = {

"alogp", "ampc_aggregation", "cns_activity", "cruzain_aggregation",

"fsp3", "firefly_luciferase", "hba", "hbd", "molecular_weight",

"nano_luciferase", "num_heavy_atoms", "num_quaternary_carbons",

"number_of_rings", "number_of_rotatable_bonds", "polar_surface_area",

"redox_interference", "solubility_water", "thiol_interference"

}

BOLTZ_PROPERTIES = {"binding_affinity", "affinity"}

Table S2: Example prompts and parsed outputs

Prompt	Seed Molecule	Targets	Iterations	Batch Size
"Optimize aspirin for better QED"	CC(=O)Oc1ccccc1C(=O)O	QED (max)	5 (default)	10 (default)
"Find the analogs of ibuprofen with improved LogP and QED. Run for 10 iterations"	CC(C)CC1=CC=C(C=C1)C(C)C(=O)O	LogP (max), QED (max)	10	10 (default)
"Starting from O=C(NCc1ccccc1OC1CCC1)c1ccc2[nH]ncc2c1 Improve binding affinity to Q92731"	O=C(NCc1ccccc1OC1CCC1)c1ccc2[nH]ncc2c1	Binding affinity (min). Structure is fetched from UniProt	5 (default)	10 (default)

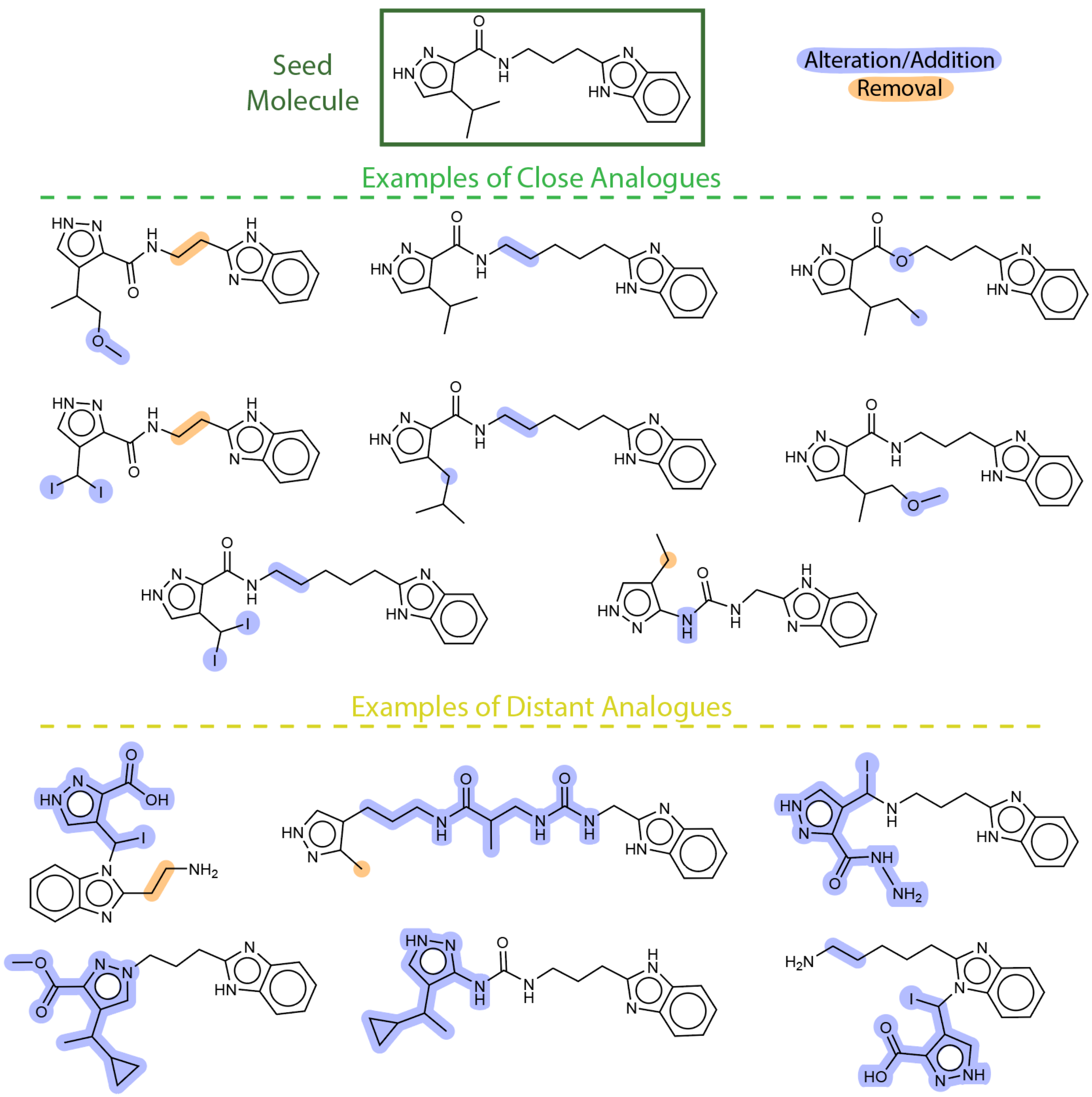

Figure S1: A sample starting compound and enumerated analogs made by HEALER software. We show samples of close and distant analogs based on the starting compound.

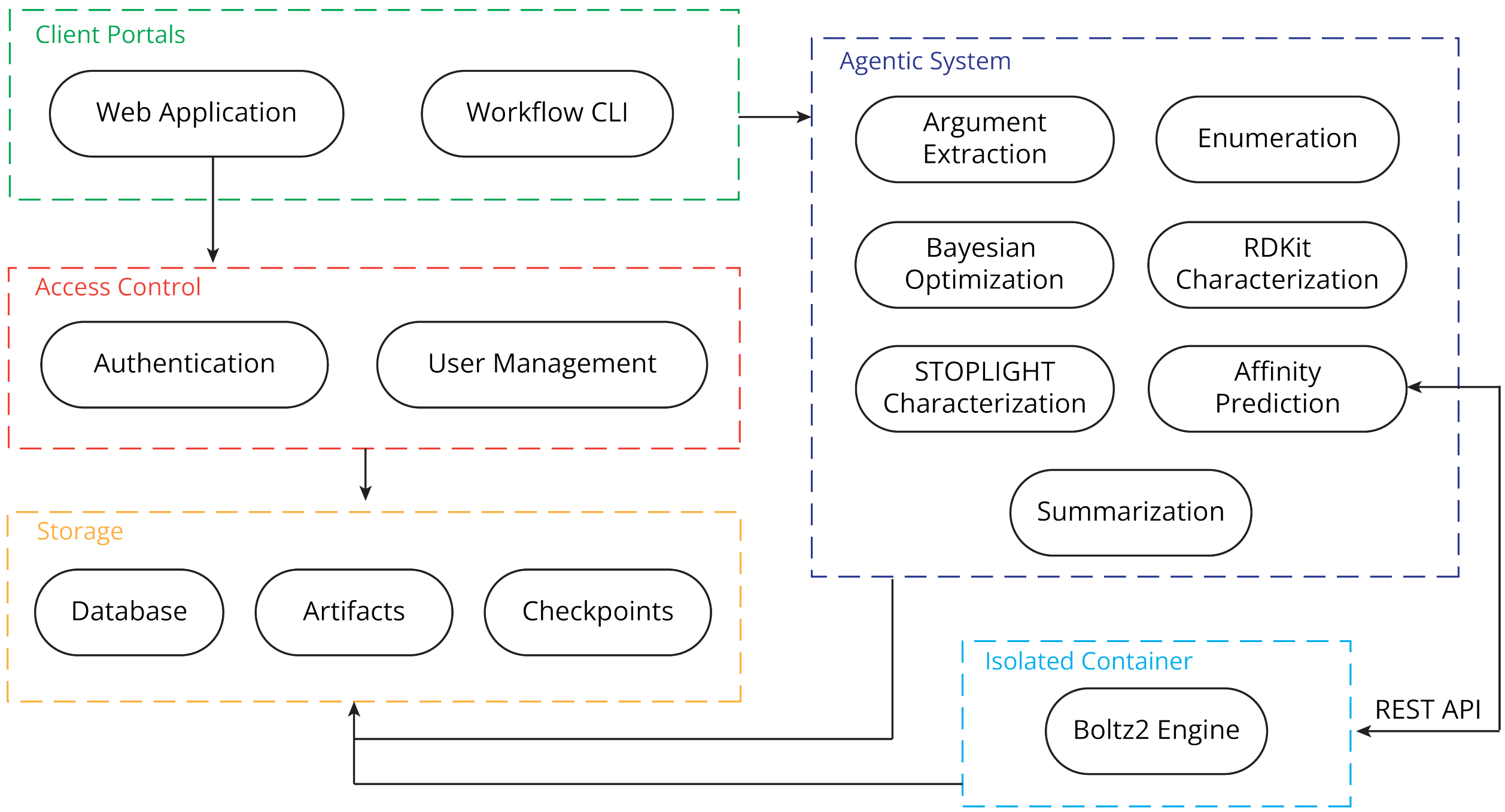

Figure S2: Architecture of SABLE's agentic system. SABLE features user-facing web and command-line interfaces through authenticated client portals, with access control, user management, persistent storage, artifacts, and checkpoints supporting reproducible workflows. The agentic system coordinates task decomposition and execution through argument extraction, molecular enumeration, Bayesian optimization, QSAR/ADME/Tox characterization, affinity prediction, and summarization nodes. Compute intensive calculations like affinity predictions are containerized in a GPU-enabled server and communicate with other nodes via a REST API.

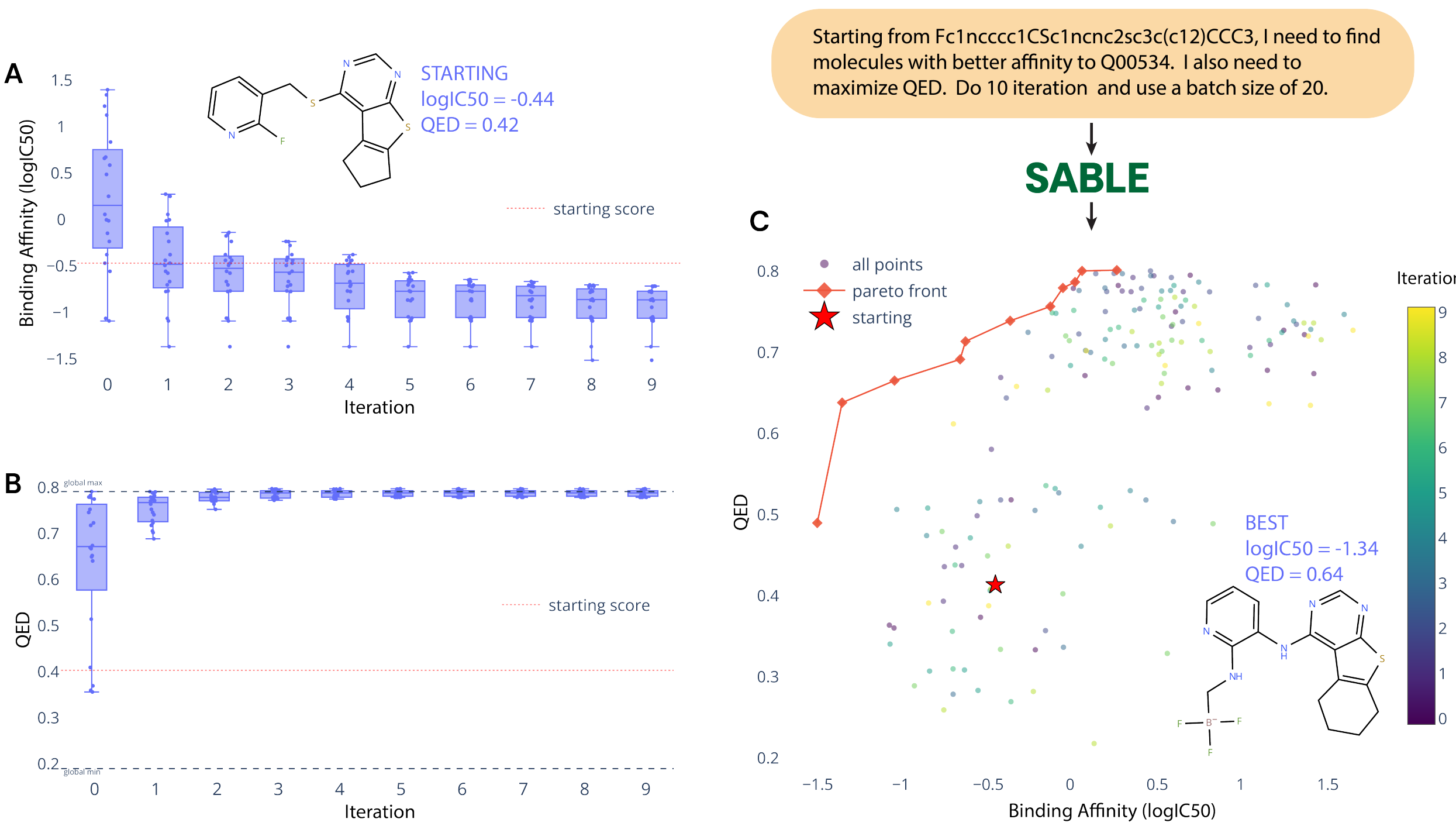

Figure S3: The dual-objective optimization of binding affinity and QED. (A) Each boxplot shows the distribution of the binding-affinity (logIC50) of the best 20 compounds selected and characterized so far in the campaign. The structure of the starting molecule with QED of 0.42 is shown. We find analogs that substantially improve the binding affinity from -0.44 to -1.48 after eight (8) iterations. (B) Iterative improvement of QED in the dual-objective campaign with the starting compound shown in the red-dotted line. We show that molecules with the best QED are found after the second iteration of the campaign. (C) The Pareto frontier showing a trade-off between binding affinity and drug-likeness. The best Pareto compound that satisfied our trade-off with an affinity score of -1.34 and QED of 0.64 is shown.

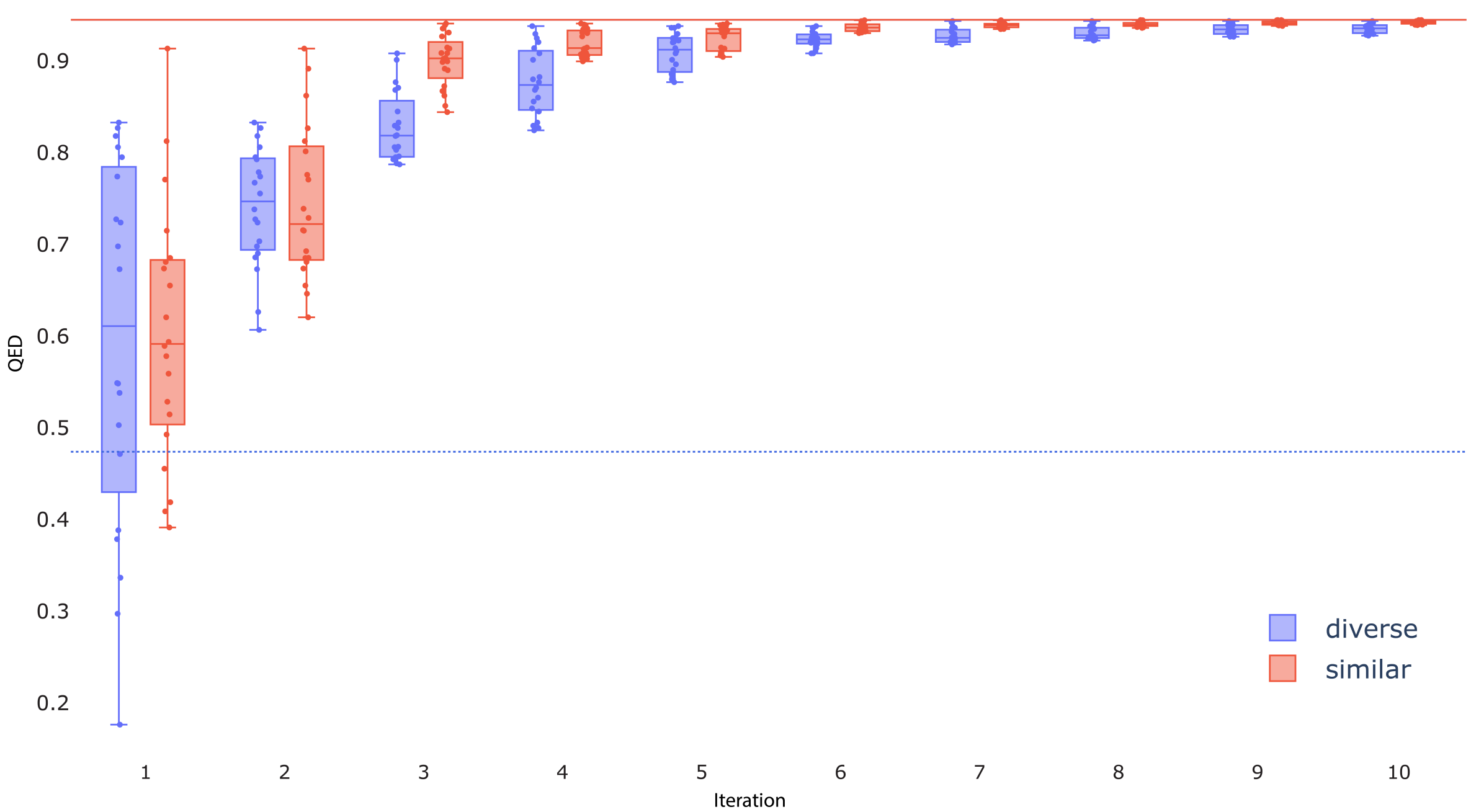

Figure S4: Optimization benchmark to determine the global maximum in a 1 million compound size library. Boxplots show the distribution of QED for the best 20 molecules obtained over ten iterations. The search space generation used a mix of two strategies: diverse and similar sampling of 1 million compounds. The blue dotted line indicates the QED of the starting compound, while the red solid line is the global maximum QED. The optimization routine finds the best compound in three iterations for the similar search space and 7 iterations for the diverse search space.

Supplementary References

(S1) Ament, S.; Daulton, S.; Eriksson, D.; Balandat, M.; Bakshy, E. Unexpected Improvements to Expected Improvement for Bayesian Optimization, 2025.

(S2) M. Bran, A.; Cox, S.; Schilter, O.; Baldassari, C.; White, A. D.; Schwaller, P. *Nature Machine Intelligence* **2024**, *6*, 525–535.

(S3) Boiko, D. A.; MacKnight, R.; Kline, B.; Gomes, G. *Nature* **2023**, *624*, 570–578.

(S4) Sprueill, H. W.; Edwards, C.; Agarwal, K.; Olarte, M. V.; Sanyal, U.; Johnston, C.; Liu, H.; Ji, H.; Choudhury, S. ChemReasoner: Heuristic Search over a Large Language Model's Knowledge Space Using Quantum-Chemical Feedback, 2024.
